# Analysis of Body Composition and Food Habits of Spanish Celiac Women

**DOI:** 10.3390/nu7075234

**Published:** 2015-07-08

**Authors:** Itziar Churruca, Jonatan Miranda, Arrate Lasa, María Á. Bustamante, Idoia Larretxi, Edurne Simon

**Affiliations:** Gluten Analysis Laboratory of University of the Basque Country, Department of Nutrition and Food Science, University of the Basque Country (UPV/EHU), Vitoria-Gasteiz 01006, Spain; E-Mails: itziar.txurruka@ehu.es (I.C.); jonatan.miranda@ehu.es (J.M.); arrate.lasa@ehu.es (A.L.); marian.bustamante@ehu.es (M.A.B.); idoia.larrechi@ehu.es (I.L.)

**Keywords:** celiac disease, gluten-free diet, diary recommended intake, food habit, body composition

## Abstract

The purpose of the present work was both to analyze composition of Spanish celiac women and to study the food habits and gluten-free diet of these celiac patients, in order to determine whether they achieve a balanced and healthy diet as well as to highlight nutritional qualitative and/or quantitative differences. 54 adult celiac women (34 ± 13 years) took part in the six-month study. Height, weight and body composition were measured. An analysis of energy consumption and of the macronutrient distribution of their diet was carried out. Their fulfillment of micronutrient intake recommendations was verified. Participants showed a Body Mass Index of 21.6 ± 2.4 kg/m^2^. Energy Intake was slightly lower than the Dietary Reference Intakes. Excessive protein apart from over-consumption of fat was observed. More than three quarters of participants consumed meat in excess. Carbohydrate consumption along with that of fiber was below recommended levels. Vitamin D, iron, and iodine had a low percentage of recommendation compliance. In general, participants followed the recommendations of dairy products and fruit intake whereas vegetable consumption was not enough for the vast majority. We conclude that although the diet of celiac women does not differ much from the diet of general population, some considerations, such as reducing fat and protein consumption and increasing fiber intake, must be taken into account.

## 1. Introduction

Celiac disease (CD) is one of the most common chronic intestinal diseases in Europe and is defined as a permanent intolerance to gluten proteins. This intolerance is maintained throughout a lifetime and is presented in genetically predisposed subjects. Its aetiology is unknown but genetic, environmental (gluten) and immunological factors contribute to its development [1]. Its estimated prevalence in Europeans and their descendants is 1%, this being more frequent in women in a 2:1 ratio [2]. Moreover, there is a significant percentage of patients who remain undiagnosed [2].

The only effective treatment for celiac disease is a strict lifelong gluten-free diet (GFD). To meet this challenge, it is essential to control the production of foods and dishes for celiac people, in order to guarantee the absence of these proteins in them. Catassi *et al.* [3] demonstrated that the ingestion of small amounts of gluten, as little as 50 mg of gluten per day over three months, can cause important damage in the intestinal mucosa. In this context a GFD with the threshold of 20 mg/kg ensures an intake of less than 50 mg/day of gluten and provides a sufficient safety margin [4,5].

Apart from gluten control, the correct suitable GFD must be nutritionally balanced too; it must fulfil all the energy and nutrient requirements and be sufficient to meet the nutritional needs of each person and prevent deficiencies. The possibility that excessively restricted cereal consumption as a solution to avoid gluten intake becomes a low carbohydrate (CHO) intake with an excess of fat and protein cannot be discarded. Additionally, the American Dietetic Association mentions in its guide for celiac patients [6] the possible consequences of complying with a GFD. According to this guide, following this diet could imply a low intake of carbohydrates, iron, folate, niacin, zinc, and fiber. Other authors also indicate that consumption of refined grains, processed as gluten-free products, entails a lower intake of vitamin B group, vitamin D, and calcium [7].

It must be taken into account that the prevalence of some diseases such as cardiovascular diseases is associated with low fiber and high saturated fat intake. Similarly, anaemia, related to a lack of iron and folic acid, and osteoporosis, associated with a lack of calcium and vitamin D, are closely linked with common symptoms of celiac patients [8,9].

As far as we know no data exist concerning the nutritional adequacy of a GFD for celiac women in Spain. In this context, the main aim of the present work was to evaluate body composition and the nutritional composition of the GFD followed by adult celiac women, as well as to compare it with the international recommendations, in order to highlight qualitative and quantitative nutritional differences. Furthermore, we found it relevant to describe the food intake and habits of celiac women in comparison with Spanish women in the general population.

## 2. Experimental Section

### 2.1. Participants and Procedure

Gluten and Food Safety is a prospective SUSFOOD study conducted in the Basque Country (Spain). The present study makes use of data from the celiac women cohort recruited in 2011 from three regions in the north of Spain (Alava, Gipuzkoa, and Bizkaia). All celiac patients were members of the Basque Country Celiac Society, with a confirmation of CD diagnosis (intestinal biopsy and/or serological test). Participants had all been compliant with the GFD for at least one year (mean length in years ± SD: 23 ± 11) and were followed up on for this analysis until April 2012 (for six months). 54 celiac women and older than 16 years took part in the study (mean age ± SD: 34 ± 13). All women claimed to be in remission from clinical symptoms. Exclusion criteria included a history of cardiovascular disease or diabetes, pregnancy, hyperthyroidism/hypothyroidism, total cholesterol levels > 300 mg/dL, levels of triglyceride > 300 mg/dL and blood pressure level > 140/90 mm Hg. All participants received verbal and written information about the nature and purpose of the survey, and all gave their written consent for their involvement in the study. This study was approved by the Ethical Committee of the University of The Basque Country (CEISH/76/2011).

Each subject underwent anthropometric parameters and dietary habits record for nutritional status assessment.

### 2.2. Anthropometric Measurements

Body weight (±10 g) was measured after voiding using a digital integrating scale (SECA 760, SECA, Hamburg, Germany). Height was measured to the nearest 5 mm using a stadiometer (SECA 220, SECA, Hamburg, Germany).

For each subject body mass index (BMI) was calculated as weight (kg)/height (m)^2^. The BMI values were categorized according to the World Health Organization (WHO) criteria as follows: Below 18.5 kg/m^2^ as underweight, 18.5–24.9 kg/m^2^ as normal weight, 25–29.9 kg/m^2^ as overweight and ≥30 kg/m^2^ as obese [10].

### 2.3. Body Composition and Energy Expenditure

Body composition (fat mass and fat-free mass) was estimated with a direct segmental multiple-frequency bioelectrical impedance analysis method (Inbody 230; Biospace, Seoul, Korea). Two skin electrodes were placed on the feet and two electrodes on the hands. According to the standard procedure, whole-body resistance and reactance were measured. For all subjects, fat mass and fat-free mass were evaluated from total-body impedance (Z). Energy expenditure was calculated using Harris-Benedict formula and applying 1.5 factor for mild/light physical activity.

Regarding the percentage of body fat, the guidelines of Gallagher *et al.* were used as reference [11].

### 2.4. Dietary Assessment

24 h food recall (24HR) of three days and a food frequency questionnaire (FFQ), previously described [12], were kept for each patient with food portions and amounts determined by using photographs of rations and sizes described in Photo Album food [13]. Trained nutritionist-dieticians carried out the 24HRs, two on weekdays and one at the weekend. Nutrient intake was calculated by using a computerized program system (AyS, Software, Tandem Innova, Inc., Huesca, Spain). The nutrient content data of the specific gluten-free products manufactured for celiac people were collected from the manufacturers and added into the food composition database of the program before calculations. As gluten-free product labels did not indicate micronutrient content (vitamins and minerals), an estimation with homologous gluten-containing products was carried out [12]. Dietary reference intakes (DRI) for Spanish population issued by the Spanish Societies of Nutrition, Feeding and Dietetics (FESNAD) in 2010 were taken as references for the interpretation of the 24HR [14]. Other Recommended Dietary Values such as Institute of Medicine (IoM) were taken into consideration [15].

In the case of FFQ, Spanish Society of Community Nutrition (SENC) recommendations were used for the correct interpretation of the results [16]. The energy, nutrient and food intakes of celiac women were compared to nutritional data obtained from a Spanish reference women population in ENIDE [17], a nutritional survey carried out in 2011, at the same period of time as the present work.

ENIDE study is representative at national level of the adult population. It was based on a random selection of more than 3323 individuals, providing a level of confidence of 95% and an accuracy of ±1.8%. The survey was conducted on 1589 men and 1734 women aged between 18 and 65 years old. The methodology used 24 h recall, daily food over three random days, and a food frequency questionnaire [17].

### 2.5. Mediterranean-Diet Score

Adherence to the Mediterranean diet was measured by the Mediterranean-Diet Score (MDS) [18]. The diet score varied from 0 (low quality diet) to 9 (high quality diet). With regard to fruits and nuts, vegetables/potatoes, legumes, fish, and cereals as well as the component ratio between monounsaturated fatty acids and saturated fatty acids, a value of 1 was assigned to celiac women whose consumption was equal to or higher than the median value, and 0 to the others. For meat and meat products and dairy products, a value 1 was assigned to celiac women whose consumption was less than the median, and 0 to the others. Although seven participants were under 18 years, taking into account that they consume alcohol, these criteria were also computed for MDS.

### 2.6. Statistical Analysis

Statistical analyses of our results were performed by using the IBM SPSS statistical program 19 (IBM Inc., Armonk, NY, USA). The results for continuous variables are given as the arithmetic mean ± SD and the range. The results for non-continuous variables are given as the frequency and the percentage. Statistical analyses were performed with Student’s or Welch’s *t* test and *F*-Snedecor test. *p* values < 0.05 were accepted as significant.

## 3. Results

### 3.1. Anthropometric and Body Composition Measurements

Main anagraphic data and anthropometric/body composition measurements of CD women are shown in Table 1. The BMI were within normal in 81.5% of cases, and there were only six people with low weight and four cases of overweight women. None of them were obese. Accordingly, the majority of adult women had a normal fat percentage [11].

**Table 1:** Characteristic of celiac participants included in survey.

Characteristic	Women
*n*	54
Age (year)	34.4 ± 12.9
Mean duration of GFD ( year)	10.9 ± 8.5
Height (cm)	164 ± 6
Weight (kg)	57.9 ± 7.3
Body Fat %	27.1 ± 6.9
**Body-mass index**	
Mean (kg/m^2^)	21.4 ± 27
Underweight <18.5—no. (%)	6 (11.1)
Normal 18.5–24.9—no. (%)	44 (81.5)
Overweight 25–30—no. (%)	4 (7.4)

Notes: Values are means ± SD; SD, standard deviation; no, number of subjects; GFD, gluten free diet.

### 3.2. Dietary Intakes

#### 3.2.1. Energy, Macronutrients and Fiber

**Figure 1:**
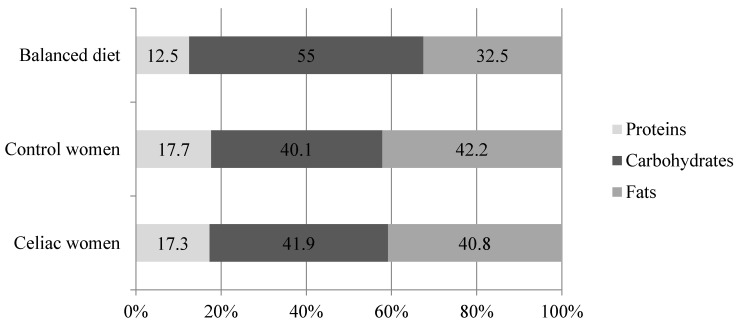
Energy percentage from each macronutrient in celiac (*n* = 54) and Spanish women—control women (*n* = 1734) (ENIDE study, representative at national level of the adult population) compared to the recommended percentage in a balanced diet proposed by the Federation of Spanish Societies of Nutrition and Dietetics (FESNAD).

The average energy intake of celiac women (Table 2) was in good accordance with their estimated energy expenditure (1904 ± 161 kcal) but below that of the DRI’s [14]. Although this was also observed in women from the ENIDE study, the energy intake of celiac patients was significantly lower (Table 2). When comparing energy sources among groups, similar macronutrient intakes were found between celiac and Spanish women—control women groups (Figure 1).

Regarding proteins, this nutrient intake represented 17.3% of total energy intake, which was similar to the data observed in the Spanish survey (Figure 1), therefore it was consumed in excess in both celiac and general women—control women populations. Animal protein intakes were the main contributor (69%) to the total protein consumption. Dairy products provided nearly 20% of total protein intake whereas meat and meat products, fish and eggs provided nearly 50%.

Carbohydrate consumption was enough to cover the minimum established as DRI (130 g/day) by FESNAD (Table 2). With regard to lipids, the percentage of this macronutrient markedly exceeded the recommendations (Figure 1). In fact, almost all the celiac women reached a fat consumption which represented over 30% of total energy. In order to evaluate the dietary fat quality, the lipid profile was calculated. In general terms, saturated and unsaturated fatty acids related ratios were reached, as was the case in the ENIDE survey (Table 2) [19].

Celiac women consume small amounts of dietary fiber. Indeed, 43% of celiac women did not reach 15 grams per day (Table 2). Compared to Spanish women—control women from the ENIDE survey, the celiac group consumed a significantly lower daily amount of fiber (Table 2). Low fiber intake was more frequent in young adult celiac women; the mean fiber consumption in the 16–44 years celiac women (*n* = 41) was 15.6 ± 5.5 g/day, women older than 45 years (*n* = 13) consumed on average 19.0 ± 5.1 g/day (*p* = 0.005).

**Table 2:** Energy and nutrient distribution in celiac and Spanish women.

	Celiac Women ( *n* = 54)	Spanish Women—Control Women (*n* = 1734)	*p*
Energy (kcal)	1847 ± 362	2038 ± 655	0.003
Protein (g)	79.2 ± 16.6	88.0 ± 37.8	< 0.001
Carbohydrate (g)	192.3 ± 40.7	199.7 ± 75.9	0.002
Fat (g)	84.6 ± 23.0	93.2 ± 35.6	0.022
(PUFA+MUFA)/SFA	2.1	2.0	-
PUFA/SFA	0.49	0.49	-
Cholesterol (mg)	324 ± 137	336 ± 151	0.544
Fiber (g)	16.4 ± 5.6	18.9 ± 10.1	0.002

Notes: Values are means ± SD; Spanish Adult women data were taken from the Spanish dietary nutritional assessment (ENIDE study, representative of the adult population at national level); SD, standard deviation; PUFA, polyunsaturated fatty acids; MUFA, monounsaturated fatty acids; SFA, saturated fatty acids.

#### 3.2.2. Micronutrients

**Figure 2:**
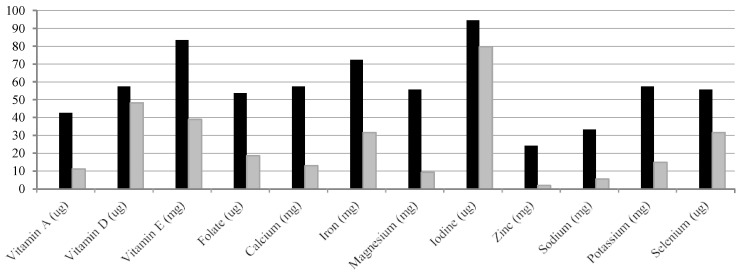
Percentage of celiac women who do not achieve dietary reference intake of the vitamins and minerals proposed by the Federation of Spanish Societies of Nutrition and Dietetics (FESNAD). ■ Do not fulfilled completely (100%) dietary reference intakes; ■ with a fulfillment of dietary reference intakes in the range of 99%–67%.

Of the 19 micronutrients analyzed, vitamin D, vitamin E, folate, iodine, iron, calcium, and selenium had an especially marked low compliance and these deficiencies were also similar to those found in general population (Table 3). None of the celiac women showed low intakes of vitamin B12 (Table 3). According to FESNAD recommendations, iodine, vitamin D, and vitamin E deficiencies were highly presented because 80%, 48% and 39%, respectively, of the celiac women did not accomplish 2/3 of DRI of these nutrients (Figure 2). Also, nearly 1 in 3 women did not achieve 2/3 of the iron and selenium DRIs (Figure 2). With respect to folate, calcium and vitamin A, around 18%, 13% and 11% of celiac were below 2/3 of recommended levels (Figure 2).

Comparing these with the Spanish general population survey—control women, micronutrient deficiencies follow a very similar scheme/ pattern (Table 3). Statistical differences were found in some of them, such as vitamin E and niacin as well as magnesium and selenium minerals, which were lower in celiacs (Table 3). On the other hand, riboflavin, vitamin B6, and folate were noticeably higher in the celiac group (Table 3).

**Table 3:** Micronutrient intake in celiac and Spanish women compared to the FESNAD’ and IoM’ recommendations.

	Celiac Women (*n* = 54)	Spanish Women—Control Women (*n* = 1734)	*p*	DRI FESNAD (2010) [14]	DRI: RDA and AI IoM (2011) [15]
Vitamin A (μg)	819 ± 556	723 ± 323	0.001	600	700
Thiamin (mg)	2.1 ± 3.5	1.8 ± 4.9	0.056	1	1.1 ^k^
Riboflavin (mg)	2.1 ± 1.5	1.4 ± 3.2	0.001	1.3 ^a^	1.1 ^k^
Vitamin B6 (mg)	2.1 ± 0.8	1.7 ± 3.7	< 0.001	1.2 ^b^	1.3 ^b^
Vitamin B12 (μg)	8.1 ± 5.6	6.1 ± 4.9	0.230	2	2.4
Vitamin C (mg)	153 ± 65	133 ± 80	0.180	60 ^c^	75
Vitamin D (μg)	4.9 ± 4.0	3.7 ± 3.7	0.331	5	15
Vitamin E (mg)	11.2 ± 3.8	13.4 ± 7.0	0.003	15	15
Niacin (mg)	26.4 ± 11.4	39.4 ± 39.7	< 0.001	14	14
Folate (μg)	373 ± 556	266 ± 113	< 0.001	300	400 ^j^
Calcium (mg)	897 ± 264	835 ± 293	0.274	900 ^d^	1000 ^d^
Iron (mg)	14.5 ± 5.0	13.7 ± 6.2	0.162	18 ^e^	18 ^e^
Magnesium (mg)	297 ± 92	354 ± 126	0.011	300 ^f^	320 ^f^
Iodine (μg)	78.7 ± 38.7	84.8 ± 47.3	0.187	150	150
Phosphorus (mg)	1223 ± 314	1295 ± 380	0.191	700 ^g^	700 ^g^
Zinc (mg)	9.2 ± 4.0	8.7 ± 3.3	0.156	7 ^g^	8
Sodium (mg)	1916 ± 802	2349 ± 810	0.505	1500	1500 ^l^
Potassium (mg)	2950 ± 806	2858 ± 827	0.485	3100 ^h^	4700
Selenium (μg)	48.2 ± 19.3	53.7 ± 28.9	0.031	55 ^i^	55

Notes: Values are means ± SD; Spanish Adult women data were taken from the Spanish dietary nutritional assessment (ENIDE study, representative of the adult population at national level); SD, standard deviation; DRI, dietary reference intake; RDA, recommended dietary allowances; AI, adequate intake; FESNAD, Federation of Spanish Societies of Nutrition and Dietetics; IoM, Institute of Medicine; ^a^ Riboflavin, 1.2 mg for 16–19 range and >60 years women; ^b^ Vitamin B6, 1.3 mg for 16–19 years old women (FESNAD), 1.2 mg for 16–18 years and 1.5 mg for >50 (IoM); ^c^ Vitamin C, 65 mg for 16–18 years (IoM) and 70 mg for >60 years (FESNAD); ^d^ Calcium 1000 mg (FESNAD) or 1300 mg (IoM) for 16–19 years range and 1000 mg >50 years women (FESNAD) or 1200 mg (ioM) for >50 years; ^e^ Iron, 15 mg for 16–19 years, 50–59 years and 10 mg for >60 years (FESNAD) or15 mg for 16–18 years and 8 mg for >51 years (IoM); ^f^ Magnesium 360 mg for 16–18 years, 310 mg for 18–30 years, 320 mg for >60 years (IoM); ^g^ Phosphorus and Zinc, 800 and 8 for 16–19years (FESNAD) or 1250 mg and 9 for 16–18 years (IoM); ^h^ Sodium, 1300 mg for >50 years women; ^i^ Selenium, 45 μg for 16–19 years (FESNAD); ^k^ Thiamin and Riboflavin: 1.0 mg for 16–18 years; ^l^ Sodium, 1300 mg for 51–70 years and 1200 mg for >70 years (IoM).

### 3.3. Food Consumption Frequency

**Figure 3:**
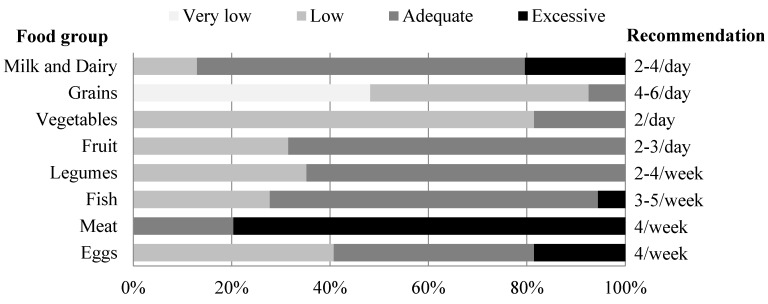
Compliance of food frequency consumption of celiac women by servings per day or week, according to Spanish Society of Community Nutrition (SENC).

Main food group consumption is summarized in Figure 3. The cereal consumption data indicated that only one out of ten celiac people ate the minimum of four recommended servings per day. Moreover, nearly half the celiac women (48%) consumed a very small amount of cereals (fewer than two servings) per day. Nevertheless, three day 24HR questionnaires showed that grains and cereal derivatives provided 23% of total energy intake and that these were the main source of CHO (37.9%). Cereal derivatives consumed usually took the form of naturally gluten-free grains and the incorporation of gluten free rendered cereals (GFP) into GFD was quite low. Specifically gluten-free products formed only 3% of total energy intake and contained only about 11 g of the 73 carbohydrate g provided by the cereal group.

One third of celiac women did not consume the minimum recommended two servings of fruit daily. In relation to the intake of vegetables, only 18.5% of celiacs consumed the daily minimum of two servings. Even weekly, the vast majority of participants, 70%, did not reach vegetable consumption recommendations (10 portions/week) [16].

As far as food of animal origin is concerned, 87% of celiac patients consumed two or more serving daily of milk or dairy products. Almost 40% of celiac patients reached the weekly recommended egg consumption by SENC. More than three quarters of participants had an excessive consumption of meat and meat products. Additionally, half this amount was fatty meat/red meat (pork, beef, lamb for instance). In the case of fish, its consumption was sufficient in two out of three adult celiacs and very few consumed it in excess. This food group (meat, fish and eggs) supplied 18% of total energy, and nearly 50% of total protein intake, whereas milk derivatives contributed 14% of total energy and 20% of total protein.

65% of celiac women usually included two or more portions of legumes and, on average, daily intake was about 20 g. Chickpeas, beans and lentils were the preferred pulses, making up 3% of the total daily energy intake.

With respect to other energy sources such as vegetable oils, sugar, chocolate, alcohol and/or pastries, most participants consumed them fairly correctly (data not shown). In general, this population chose olive oil as its fat source, a positive aspect that contributed to maintaining the high proportion of monounsaturated fatty acid in the diet.

### 3.4. Score for Adherence to Mediterranean Diet

The MDS showed that diet followed by celiac women was intermediate in quality, in terms of adherence to the Mediterranean pattern. The mean MDS for all studied participants was 4.6 ± 1.8.

## 4. Discussion

Dietary treatment of celiac disease involves certain dietary restrictions that can limit the nutritional status of celiac people and make the implementation of the recommendations for a balanced diet difficult. In a previous study carried out by our research group, females and males were collected in order to analyze their GFD and to carry out a nutritional comparison between gluten-free diets and diets containing equivalent products with gluten [14]. Surprisingly, women and men showed different patterns of eating habits. Following a GF diet in women resulted in a lower protein intake and in a higher fat intake when comparing the same diet with equivalent products containing gluten. However in men these differences were not observed. Taking these results into account, a more in-depth study should be carried out in order to analyze the nutritional status of celiac women following a GFD. Thus, in the present research, we evaluated this situation in 54 celiac women from the Basque Country (from Alava, Gipuzkoa, and Bizkaia regions in the north of Spain), in order to point out any differences between celiac women and women in the general population.

The anthropometric study conducted with celiac women indicated that only less than 6.5% were overweight and that there were no cases of obesity. In good accordance with these results, Bardella *et* *al.* found a lower BMI index in celiac people than in the general population [20]. Our results were also lower than those corresponding to Spanish women—control women overall [21]. There are several reasons that could justify this fact: (1) the celiac disease pathological situation could provoke a low bioavailability of nutrients as was reported by others in newly diagnosed celiacs [22]; (2) these patients were more concerned about their dietary habits than the general population and therefore there is a lower percentage or incidence of overweight and obesity.

With regard to the first issue, it is hard to believe that malabsorption related to celiac disease could be an explanation, considering that participants in our study had followed a long-term GFD (10.9 years). However, it is true that in the case of the adult celiac population complete normalization of duodenal lesions is rare [23], leading to additional nutrient deficiencies. One of the weaknesses of the present research is that no intestinal biopsy was performed prior to the study, therefore there was no possibility of ensuring the histological remission of celiac disease, which makes it difficult to discount if any of the celiacs studied might have more deficiencies or not. Nevertheless, the purpose of the study was to define whether GFD itself could provoke nutrient deficiencies or not, regardless of histological remission. Despite its limitations, the information extracted from the present research could answer why celiac patients in biopsy-proven remission and who adhered to a strict gluten-free diet for years were prone to the development of various vitamin deficiency states [24].

The second reason itself could also be considered as one possible limitation of this work, due to the fact that research study participants usually show more interest in the topic of nutrition. Taking into account that the present study was voluntary and was not done randomly, the self-selection bias was inevitable. However, it is noteworthy that our results were consistent with those obtained in a study conducted with an American celiac population. Zipser *et al.* [25] found a prevalence of overweight and obesity of 14% and 4% respectively in American Celiac population, percentages which were a long way from the prevalence of overweight and obesity in the United States. Other recent research carried out in Finland also showed that although the mean BMI is significantly increasing in Western countries and a similar trend is described for celiac patients, a more favourable BMI [26] is found in the latter population.

Another important aspect in assessing the nutritional status of celiac was the dietary intake evaluation. Nowadays there are no databases that include specific composition of GFP. Furthermore, gluten-free product labels do not indicate micronutrient (vitamin and mineral) content. As stated before, in the present study an estimation using homologous gluten-containing products was carried out. This strategy has also been used by other authors, publishing some results in good accordance to ours [27].

According to data obtained from the three day records, the macronutrient imbalance in the celiac population was quite predictable considering that it also exists in the general population. In fact, the national survey of dietary intake of Spain (ENIDE) showed that a similar percentage of energy intake comes from protein, and a slightly larger one from fats (42.2%) in the Spanish population—control women [17]. These similarities between GFD energy distribution and that of the general population have been reported in other studies conducted in Europe [28,29].

It is important to remark that the low consumption of CHO detected in celiac women by our study, was accentuated in general Spanish population—control women. Furthermore, it must be considered that the percentage contribution of carbohydrates to total energy has steadily decreased in recent years. This fact is due at least in part to reductions in cereal and grain intake. The ENIDE survey indicated that 39% of total energy from CHO came from cereal, and that this food group contributed 17% (394 kcal) of total energy, which is comparable to the data reached by our celiac group (37,9% of total carbohydrate and 23% of total energy; 417 kcal) [17]. Both sets of data, the ENIDE survey and our study, showed cereal and grain consumption below Spanish data registered in 2006 and in previous years [30].

With the outcomes obtained from celiac women *vs.* general women population—control women it could be hypothesized that the dietary habits of celiac women were healthier than those of the general population. Zuccotti *et al.* [29] compared dietary intake of celiac children to a control group, suggesting a similar conclusion. Food frequency questionnaires of that study revealed a difference in eating habits. Indeed, celiac children consumed more bread and rice, accompanied by greater gluten-free product consumption than controls [29]. Nevertheless, it is necessary to point out that celiac women from our research did not consume such a large amount of commercially available gluten-free foods as those children did.

As suggested by Subar *et al.* [31], in the general United States population grain foods contribute a large percentage to the adult daily intake of several nutrients including thiamine, riboflavin, niacin, folate, iron, and fiber [31]. Therefore, non-consumption of the recommended amount of whole grain products could have important implications for dietary intakes of B-vitamins, iron, and fiber.

With regard to our results, the intake of dietary fiber in a GFD was a long way from the recommended amount and it was even much lower than Spanish women`s consumption—control women. This fact was not surprising, taking into account that GFD and GFP are often formulated and produced in low fiber forms to avoid the risk of gluten cross-contamination [32]. Unlike our results, in a recent study conducted with the German celiac population, dietary fiber intake was very similar to that of the general German population [33], which suggests that perhaps commercial gluten-free food contains added fiber in that country. However, another study conducted with Spanish families [34] indicated that the average fiber consumption is about 16.4 g/day, which would be a similar amount to that found in the celiac women population. Therefore it could be postulated that adult celiac women share nutritional goals with the rest of the Spanish population, at least for this component of the diet.

As micronutrients are concerned, if we compare the results of the celiac population with Spanish general population—control women, the data are similar to those reported in the ENIDE study [17]. Vitamin D and iodine represent the most important deficiencies in the celiac women of this study [14].

The compliance rate for recommended vitamin D consumption, both for the celiac as well as for the general population, is around 70%–80% of RDI. The lack of vitamin D can alter the normal metabolism of the bone, which can lead to rickets in children or osteoporosis or osteomalacia in adults [35]. In fact, numerous studies indicate that a suitable intake of calcium is crucial in the celiac group [36,37]. In this sense, the data showed that 57% of celiacs do not obey the FESNAD DRI for calcium [14]. A review of the topic suggested that up to 75% of celiac people exhibit low bone density and increased risk of fractures [38]. What is more, a study of persistent mucosal damage and the risk of fracture in celiac disease concluded that the association between persistent villous atrophy and hip fractures implies thinner subcutaneous tissue [39]. Taking into account that our result showed that 6% of participants were underweight and deficient in vitamin D intake, the risk of fractures in our cohort is more than probable.

Similarly, as in the Spanish general population—control women, there was a deficiency of iodine in the celiac participants (94% of them demonstrated iodine deficiency according to FESNAD’s recommendations) [14]. In good accordance with the result obtained, remarkably low iodine intake was also found in Europe in 2010 for the general population [40]. Nevertheless, it is important to point out that celiac disease is accompanied by other pathologies related to thyroid function, where adequate iodine consumption is a limiting factor to avoid clinical consequences [41].

In the case of vitamin A, this micronutrient had a low rate of compliance in celiac women, even though the average intake was greater than in the Spanish general population—control women (ENIDE study) [17]. For the rest of the minerals and vitamins analyzed, recommended intakes were reached (Table 3) [14]. Although, riboflavin results show that the celiac population had doubled RDI [14], there is no evidence of adverse effects due to excessive intake of riboflavin, possibly due to a limited extent of absorption in the intestine and its rapid excretion in urine.

Vitamin 6, folate, and vitamin B12 deficiencies commonly lead to moderate elevations in total plasma homocysteine levels which, in turn, may increase the tendency to develop occlusive venous and arterial disease in both celiac people and the general population [42]. In our research, the recommendations for vitamin B6, folate, and vitamin B12 were amply fulfilled by celiac women. Taking into account that it has been demonstrated that most bread, pastas, and cold cereals are not fortified with folate for instance [43], the intake of this vitamin, like that of B12 and B6, must have been compensated by other sources.

The frequency consumption questionnaire conducted revealed the daily or weekly consumed rations of different food groups. Although macronutrient distribution of the diet was similar to the rest of the Spanish population [16], this questionnaire highlighted that the food source of these nutrients was different. CHO in the general population should come from cereals, fruits and pulses/legumes, respectively. As represented in Figure 3, celiac women consumed a very low amount of cereals, consequently in the case of celiac people the pulses and legumes group had greater importance as sources of CHO. Thus, if we compare the consumption of legumes of celiac people in our research with Spanish general population [30], it is possible to point out the great difference between groups (19.3 g/day *vs.* 11.9 g/day). The strategy followed by celiac people consists in focussing on avoiding gluten in the diet, promoting the consumption of legumes instead of cereals [44].

In relation to the intake of vegetables, although only 19% of celiac adults consume the daily minimum of two servings these data are in good accordance with the rest of the Spanish adult population—control women [17]. More than three quarters of participants reported excessive (more than three servings) consumption of meat and meat products per week. Additionally, rather than celiac people consuming one serving per week of fatty meat/red meat, the data obtained revealed that half of their meat consumption was pork, beef, or lamb. Nevertheless, the daily consumption of meat by celiac patients was lower than that of the general Spanish population (110 g/day *vs.* 179 g/day) [30].

The PREDIMED trial among others, has demonstrated that following a Mediterranean Diet can be considered a sustainable ideal model for cardiovascular disease prevention [45]. According to data published in the ENIDE study, celiac women demonstrated better adhesion to the Mediterranean diet than did the general Spanish women population—control women. The percentage of celiac women with low adherence to the Mediterranean dietary pattern (less than index 4) was 30%, while in the Spanish women population—control women this reached 62%. Consequently, celiac women showed higher percentages of intermediate (indexes 4–6) and high (>6) Mediterranean-Diet Score; 56 *vs.* 31 and 13 *vs.* 7 respectively. In good accordance with the results obtained in the present work, a recent study conducted with Spanish women—control women established a relationship between higher adhesion to the Mediterranean diet and decreased risk of overweight or obesity [46].

In summary, as far as our result is concerned there are marked differences between the body composition of adult celiac and non-celiac women. Furthermore it could be postulated that energy and macronutrient intake of celiac women follows trends similar to those found in Spanish women—control women as a whole, with an imbalanced distribution of macronutrients and an inadequate consumption of certain micronutrients and fiber (Table 4).

**Table 4:** Summary of Spanish societies recommendations’ compliance concerning energy intake, obese and overweight percentage, nutrients, fiber and food consumption frequency by celiac women and Spanish women.

	Compliance/Suggestion	
	Celiac Women	Spanish Women—Control Women
Energy intake (kcal)	√	
Obese and overweight (%)	√	×/↓
Macronutrient distribution		
Protein (%)	×/↓	
Carbohydrate (%)	×/↑	
Fat (%)	×/↓	
Fiber (g)	×/↑↑	×/↑
Micronutrient intake		
Vitamin D (μg)	×/↑	
Vitamin E (mg)	×/↑	
Folate (μg)	×/↑	
Calcium (mg)	×/↑	
Iron (mg)	×/↑	
Iodine (μg)	×/↑	
Selenium (μg)	×/↑	
Food consumption frequency		
Grains	×/↑	√/=
Legumes	√/=	×/↑
Vegetables	×/↑	

Notes: ×, values do not achieve Spanish societies (FESNAD and SENC) recommendations; √ values do achieve Spanish societies (FESNAD and SENC) recommendations; ↑ it is suggested an intake increase; ↓ it is suggested an intake reduction; FESNAD, Federation of Spanish Societies of Nutrition and Dietetics; SENC, Spanish Society of Community Nutrition.

## 5. Conclusions

Even though specific nutritional education is not necessary for celiac people some specific consideration must be provided in order to improve eating habits and nutritional status among adult celiac women.
